# Genomic variants in an inbred mouse model predict mania-like behaviors

**DOI:** 10.1371/journal.pone.0197624

**Published:** 2018-05-16

**Authors:** Michael C. Saul, Sharon A. Stevenson, Changjiu Zhao, Terri M. Driessen, Brian E. Eisinger, Stephen C. Gammie

**Affiliations:** 1 Carl R. Woese Institute for Genomic Biology, University of Illinois at Urbana-Champaign, Urbana, Illinois, United States of America; 2 Department of Integrative Biology, University of Wisconsin-Madison, Madison, Wisconsin, United States of America; 3 School of Medicine, Yale University, New Haven, Connecticut, United States of America; 4 Waisman Center, University of Wisconsin-Madison, Madison, Wisconsin, United States of America; 5 Neuroscience Training Program, University of Wisconsin-Madison, Madison, Wisconsin, United States of America; National University of Ireland Galway, IRELAND

## Abstract

Contemporary rodent models for bipolar disorders split the bipolar spectrum into complimentary behavioral endophenotypes representing mania and depression. Widely accepted mania models typically utilize single gene transgenics or pharmacological manipulations, but inbred rodent strains show great potential as mania models. Their acceptance is often limited by the lack of genotypic data needed to establish construct validity. In this study, we used a unique strategy to inexpensively explore and confirm population allele differences in naturally occurring candidate variants in a manic rodent strain, the Madison (MSN) mouse strain. Variants were identified using whole exome resequencing on a small population of animals. Interesting candidate variants were confirmed in a larger population with genotyping. We enriched these results with observations of locomotor behavior from a previous study. Resequencing identified 447 structural variants that are mostly fixed in the MSN strain relative to control strains. After filtering and annotation, we found 11 non-synonymous MSN variants that we believe alter protein function. The allele frequencies for 6 of these variants were consistent with explanatory variants for the Madison strain’s phenotype. The variants are in the *Npas2*, *Cp*, *Polr3c*, *Smarca4*, *Trpv1*, and *Slc5a7* genes, and many of these genes’ products are in pathways implicated in human bipolar disorders. Variants in *Smarca4* and *Polr3c* together explained over 40% of the variance in locomotor behavior in the Hsd:ICR founder strain. These results enhance the MSN strain’s construct validity and implicate altered nucleosome structure and transcriptional regulation as a chief molecular system underpinning behavior.

## Introduction

Bipolar spectrum disorders (BSDs) are a heterogeneous group of mental health diagnoses marked by episodes of mania and depression [1]. BSDs are highly heritable [2]. Development of treatments for BSDs derived from these heritable diseases’ genetic correlates has been the goal of over a decade’s worth of genome-wide studies of mental health disorders [3]. Despite the considerable resources invested in genomic work on BSDs, a convincing molecular etiology remains elusive [3,4]. Difficulty in defining BSDs at the molecular level is attributable to complexity. Genome-wide linkage scans and association studies of BSDs contain multiple genomic findings that change depending upon the population examined [5–8], suggesting that the disease originates from heterogeneous alterations of many genes in the biological systems contributing to the BSDs phenotype. Further, the biological correlates of BSDs show relationships to other mental health disorders like schizophrenia [9]. Because of these complexities, studying BSDs necessitates a systems biology approach [10,11].

Animal models for BSDs should also utilize a systems approach. In rodents, models for BSDs split these illnesses into constituent behavioral endophenotypes reflecting mania and depression [12]. As far as we know, nobody has observed an animal model showing replicable cycling between mania-like and depressed-like behavioral states [13]. Animal models for mania fit typically into three general categories: transgenics, pharmacological manipulations, and strain variants [12].

The Madison (MSN) mouse strain is an inbred strain derived from the outbred Hsd:ICR (ICR) founder population over the course of 15 years. MSN mice are a face-valid mania model displaying a suite of behaviors associated with a manic endophenotype including locomotor hyperactivity, decreased swim immobility, increased sexual behavior, advanced diurnal rhythm, and seasonal-like alterations in locomotor hyperactivity relative to control strains [14,15]. MSN animals are consistent with a predictively valid mania model, showing the expected attenuation of mania when treated with lithium chloride or the atypical antipsychotic olanzapine [14]. Further, MSN animals show hippocampal gene expression perturbations similar to those seen in post-mortem limbic brain tissue from patients with BSDs including alterations in a pathway related to chromatin packaging [16], a finding that establishes provisional construct validity. Related strains to the MSN strain show additional alterations in chromatin packaging in the striatum [17], providing additional support for brain chromatin packaging as an important system dysregulated in these mice. The MSN phenotype is highly reproducible, showing mania-like behaviors at the same approximate magnitude across multiple generations [14–16]. Altogether, we believe the MSN strain is a novel, unique, useful mania-like model that shows many relevant biological similarities to the human disorder it models [16].

Here, we report the results of a small-scale whole exome resequencing study on MSN mice to identify potential candidate alleles, then validate a small subset of these candidate alleles in a large population using a low-throughput genotyping method. Based upon our previous gene expression and behavioral work, we predicted variants unique to the MSN strain in purinergic reception related genes, molecular clock genes, and chromatin remodeling genes [15,16]. Genomic enrichment of our gene expression work predicted that MSN animals would show perturbations in genomic regions sharing synteny with human loci linked to bipolar disorder and related mental health disorders [16]. Though these *a priori* predictions are useful for finding causative variants, exome resequencing allows for querying all protein-coding sites. Thus, our exploratory approach allowed us to query genes and gene systems with structural variants unrelated to these systems.

## Results

**Fig 1:**
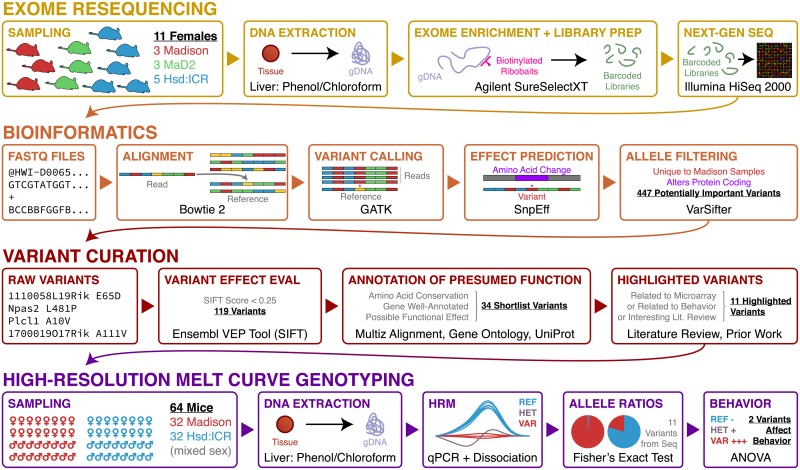
Pipeline diagram showing a schematic of the protocols used in this paper. Briefly, the results are derived from a pipeline including: exome resequencing, bioinformatics, variant curation, and high-resolution melt curve genotyping. These procedures were used to identify MSN variants that to affect behavior in the founder strain.

The experimental protocol used to identify and test interesting candidate variants within this manuscript is represented as a diagram of pipelines for exome resequencing, bioinformatics and variant curation, and high-resolution melt curve genotyping (Fig 1). Based upon the strengths and limitations of exome resequencing, we were interested in identifying three types of variants: non-synonymous single nucleotide polymorphisms (nsSNPs), which alter a protein-coding sequence at a single amino acid residue; short insertions and deletions (short INDELs), which are focal loci with a few base pairs inserted or deleted from the genome; and nonsense-mediated mRNA decay (NMD) variants, which are predicted to alter protein coding such that a surveillance pathway selectively degrades mRNA transcripts containing premature stop codons.

Exome resequencing identified 447 nsSNPs, short INDELs, and NMD variants that affected coding sequence, mismatched the reference genome, appeared to be fixed in MSN mice, and showed some variability in control strains. We filtered these variants using Sorting Intolerant from Tolerant (SIFT) scores, which predict whether an amino acid substitution affects protein function on a scale from 0 (deleterious) to 1 (tolerated) [18]. We found 119 candidate variants in 87 genes that included nsSNPs with SIFT scores ≤ 0.25, short INDELs, NMD variants, or other interesting variants (S1 Table). Querying these genes in DAVID (S2 Table) yielded significant enrichment clusters related generally to histocompatibility (DAVID enrichment score = 3.75), metal ion binding (DAVID enrichment score = 2.30), and a marginally significant enrichment cluster related to G-protein-coupled receptors (DAVID enrichment score = 1.98). We further queried these variants using UniProt and the UCSC Genome Browser for gene ontology, sequence conservation across species, variant novelty, and presumed effects on protein function, annotating each variant with our findings. Based upon this initial manual curation, we compiled a short list of 34 variants in 24 genes likely to change protein function (Table 1, variants highlighted in grey in S1 Table). Functional enrichment on these variants (S2 Table) identified a significant enrichment cluster for metal ion binding (DAVID enrichment score = 2.51).

**Table 1:** Short list protein-coding variants unique to MSN. Of the variants of interest including short INDELs or nsSNPs with SIFT scores less than 0.25, these 34 variants were the ones we believed most likely to change protein chemistry based upon exhaustive hand annotation of each variant using UniProt. All short INDELs and nsSNPs with SIFT scores less than 0.25 are included in S2 Table.

Location and Allele	Gene ID	UniProt	Consequence	Amino Acid	SIFT
chr1:39336044 T/C	*Npas2*	P97460	Missense	L481P	0.23
chr2:25363944 C/T	*Uap1l1*	Q3TW96	Missense	G300D	0.13
chr2:167036853 C/T	*Znfx1*	Q8R151	Missense	G1868S	0.00
chr3:19980665 T/G	*Cp*	G3X8Q5	Missense	C712G	0.01
chr3:19987423 G/A	*Cp*	G3X9T8	Missense	S903N	0.22
chr3:20005467 T/C	*Cp*	G3UXD2	Missense, Splice	V23A	---
chr3:20012808 C/A	*Hps3*	E9PZY1	Missense	K531N	0.16
chr3:20012845 T/C	*Hps3*	Q91VB4	Missense	N651S	0.14
chr3:20058944 C/T	*Hltf*	G3UVU1	NMD	H22	---
chr3:20076540 G/C	*Hltf*	Q6PCN7	Missense	K369N	0.20
chr3:96719304 G/A	*Polr3c*	Q9D483	Missense, NMD	T268M	0.00
chr5:31073236 G/A	*Cad*	B2RQC6	Missense	R1608H	0.00
chr5:107433141 G/A	*Lpcat2b*	Q9D5U0	Missense	R112H	0.04
chr5:110127696 C/T	*Zfp605*	E9QAH2	Missense	P227S	0.01
chr5:117555266–117555277 Deleted	*Ksr2*	M0QW59	Long Deletion	VRTPP259-263V	---
chr8:89032312 G/T	*Sall1*	Q6P5E3	Missense	A388E	0.02
chr8:93076109 A/G	*Ces1b*	D3Z5G7	Missense	Y118H	0.01
chr8:121916500 G/A	*Car5a*	P23589	Missense	T255M	0.14
chr9:13826880 C/A	*Cep57*	D6RH89	NMD	S7	---
chr9:21637471 G/A	*Smarca4*	Q3TKT4	Missense	R351Q	0.04
chr9:21835572 G/A	*Gm6484*	Q8R1L8	Missense	A19T	0.11
chr11:59000521 C/CTGG	*Obscn*	F6TJX7	Insertion	V1583PV	---
chr11:59000884 C/T	*Obscn*	H7BX05	Missense	V6941M	0.12
chr11:59076137 G/A	*Obscn*	J9JIB2	Missense	H586Y	0.02
chr11:73240601 C/G	*Trpv1*	Q704Y3	Missense	P14A	0.00
chr11:73254291 C/A	*Trpv1*	Q704Y3	Missense	D734E	0.05
chr12:112495085 C/T	*Nrac*	Q8BNX7	Missense	R28W	0.05
chr12:112497893 C/T	*Nrac*	Q8BNX7	Missense	R110C	0.12
chr12:112498037 T/C	*Nrac*	Q8BNX7	Missense	C158R	0.23
chr12:118190825 T/C	*Dnahc11*	E9Q7N9	Missense	E240G	0.18
chr12:118198712 G/A	*Dnahc11*	E9Q7N9	Missense	R41C	0.01
chr17:7772146 A/T	*Fndc1*	E9Q043	Missense	L906H	0.06
chr17:54297024 C/T	*Slc5a7*	Q8BGY9	Missense	R38H	0.08
chr17:81054224 C/CTAT	*Thumpd2*	Q9CZB3	Insertion	S191NS	---

Based upon previous behavioral work demonstrating alterations in seasonality in MSN mice [15], a variant in *Npas2* interested us greatly due to that gene’s involvement in seasonal affective disorder [19]. Brain gene expression work on MSN animals implicated transcriptional regulation as an important system dysregulated in the MSN brain [16], so we were particularly motivated to examine variants in *Hltf* (*Smarca3*), *Cp*, *Polr3c*, and *Smarca4*. Additionally, literature review showed the following genes to be compelling candidates for downstream analysis: *Ces1b* [20], *Slc5a7* [21], *Trpv1* [22], *Lpcat2b* [23], *Cad* [24], and *Nrac* [25]. Brief summaries of these criteria are included in S1 Table. We chose the variants in these genes for downstream analysis of population allele ratios in both MSN and ICR using HRM genotyping.

**Fig 2:**
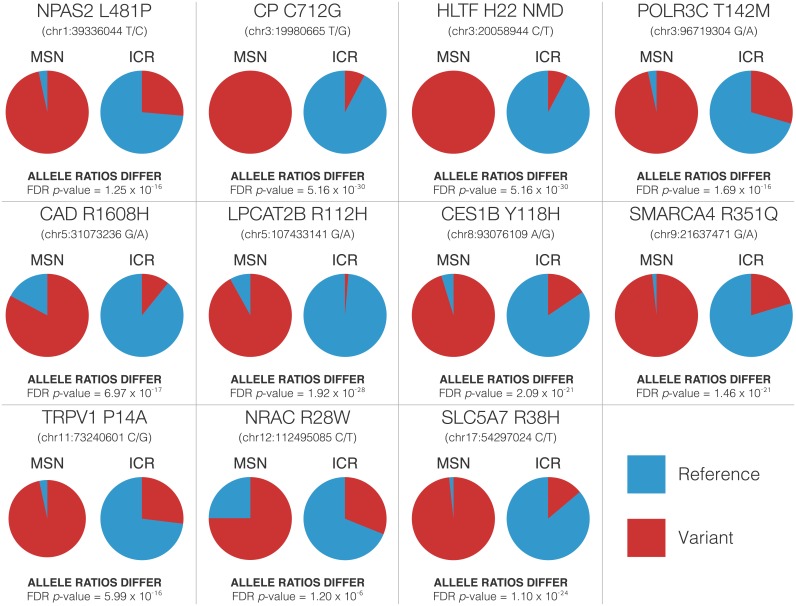
HRM population allele ratios for 11 variants of interest. The allele ratios for each these variants was assessed using HRM genotyping. Allele ratios were compared between MSN and ICR using a Fisher’s Exact test and FDR-corrected for multiple comparisons using the Benjamini-Hochberg method. All 11 comparisons were highly statistically significant, though not all allele ratios showed the near fixation necessary to establish biological significance.

Allele ratios differed significantly between MSN and ICR animals for each variant queried, though some showed a higher degree of population fixation in MSN (Fig 2). After applying false discovery rate (FDR) correction, there were no significant deviations from Hardy-Weinberg equilibrium in either strain for any variant, indicating that no chosen allele is under measurable selection in the tested generation. Because the MSN phenotype is highly reproducible and based on dozens of generations of inbreeding, variants showing less than complete fixation in *Nrac* (46.9% of MSN animal with variant alleles), *Cad* (31.2% of MSN animals with variant alleles), and *Lpcat2b* (15.6% of MSN animals with variant alleles) are likely of lower phenotypic relevance than those showing a high degree of population fixation (raw HRM results, allele and genotype ratios, and statistical tests are included in S3 Table).

**Fig 3:**
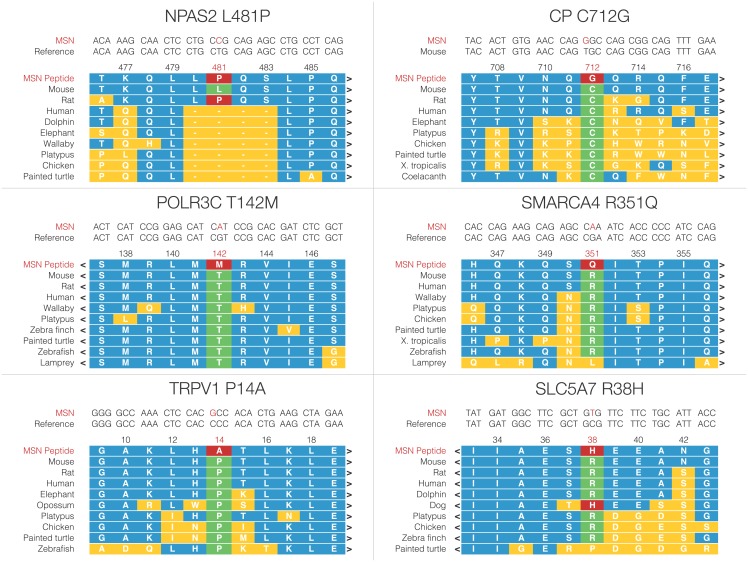
Selected interspecific alignments for six high-interest variants. Using the UCSC Genome Browser’s Multiz Alignments for selected vertebrate genomes, we visualized homology between a diverse sampling of aligned genomes for each coding variant, adding 5 amino acids on each side for context. The MSN sequence is highlighted in red while the reference at each genome is highlighted in green. The context is highlighted in blue, and any variation away from reference that is not the same as the MSN variant is highlighted in yellow. More highly conserved residues are more likely to change protein chemistry and have biological effects.

Of the variants examined in HRM, we believe six are of the highest biological interest based upon their near total fixation in MSN, consistency with previous gene expression and behavioral results, and neurobiological relevance. These variants, now referred to in their protein-coding nomenclature, are NPAS2 L481P, CP C712G, POLR3C T268M, SMARCA4 R351Q, TRPV1 P14A, and SLC5A7 R38H. We visualized these variants’ alignment to peptide sequences of multiple species using the UCSC Genome Browser’s Multiz alignment for each variant (Fig 3). For each variant allele, we generated a functional hypothesis for its effects on the mature protein’s function (Table 2).

**Table 2:** Six high interest variants with functional hypotheses. For these variants, we advanced mechanistic ideas about how they change the proteins of interest. Where possible, these hypotheses are testable, allowing for further characterization of protein chemistry in the future.

Variant	Functional Hypothesis
NPAS2 L481P	L481P is very close to human variant S471L, associated with seasonal affective disorder.
CP C712G	From structural biology on the human peptide, C712G breaks a disulfide bond.
POLR3C T268M	T268M is in a conserved site in an alpha helix. It likely changes secondary structure.
SMARCA4 R351Q	R351Q likely abolishes phosphorylation of T353.
TRPV1 P14A	P14A changes a conserved residue in an ankyrin domain of the mature channel.
SLC5A7 R38H	R38H is in the cytoplasmic domain of the mature acetylcholine transporter.

The variants in the genes *Smarca4* and *Polr3c* appeared to predict spontaneous locomotor behavior in the outbred ICR strain. In two-way ANOVAs on transformed behavioral data from previous work [15], the interaction between *Smarca4* genotype and sex significantly accounts for variance in the ICR strain’s locomotor behavior (F_1,28_ = 7.126, *p* = 0.0125, model multiple R^2^ = 0.337, model adjusted R^2^ = 0.266) and the genotype at the *Polr3c* variant alone significantly accounts for variance in the ICR strain’s locomotor activity (F_1,28_ = 9.854, *p* = 0.00397, model multiple R^2^ = 0.299, model adjusted R^2^ = 0.224). Further, a model including only the effects of the *Smarca4* genotype-by-sex interaction and the *Polr3c* genotype main effect boosts the model multiple R^2^ to 0.409 and the model adjusted R^2^ to 0.322, indicating that the two genotypes together explain more variance better than they do in isolation.

## Discussion

We found nearly fixed variant alleles in six genes that we predict will affect behavior. These genes’ products are diverse in ontology and function: NPAS2 is a transcription factor and canonical molecular clock gene [26], CP is involved in cellular iron efflux (Harris et al. 1999), POLR3C is a subunit of RNA polymerase III [27], SMARCA4 is an ATP-dependent DNA helicase with chromatin remodeling properties [28], TRPV1 is the transient receptor potential channel responding to excessive heat and capsaicin that has found recent acceptance as an ionotropic endocannabinoid receptor [29], and SLC5A7 is a neuronal choline transporter [30]. These variants are subtle, indicating that the MSN mania model’s phenotype likely results from a complex genotype involving small changes in multiple genes. Locomotor hyperactivity is difficult to selectively breed into ICR mice [31]. Because these variant alleles show low but measurable population allele frequencies in the outbred ICR strain, we believe that a polygenic origin explains the MSN mania phenotype.

These genes appear relevant to the mania-like behavioral endophenotype. Furthermore, many of these genes belong to systems previously linked to BSDs in humans. Of preeminent interest to our work is SMARCA4, whose human ortholog is among a network of gene products predicted to play a role in the differential chromatin dynamics of BSDs based upon a meta-analysis of gene expression data from postmortem brain tissue of patients [10]. The genome region and a molecular interaction network containing *Smarca4* are perturbed in our previous work [16,17]. Further, the human genome region containing the human *SMARCA4* gene sits between D19S714 and D19S586, markers with significant linkage to BSDs in two independent studies [8,32].

The particular SMARCA4 variant we identified occurs at R351, a highly conserved residue in the protein two amino acid residues away from T353. This threonine residue that was found phosphorylated in a high-throughput phosphoproteomic screening of human cells [33]. Using GPS 2.1 and NetPhos 2.0, phosphorylation prediction software packages with divergent prediction algorithms [34,35], we found that the subtle change of an arginine to a glutamine residue in the variant is predicted to abolish T353 phosphorylation. Further, SMARCA4 shows nucleosome remodeling activity in relation to RNA polymerase activity [28].

The other variants that we discuss appear to have potential relationships to mania in the literature. Variants in the human ortholog in the vicinity of NPAS2 L481 are correlated with seasonal affective disorder [19], and BSDs have high comorbidity with seasonal affective disorder [36]. Not only are cannabinoid receptors associated with mood and anxiety disorders [37], BSDs have an unusually high comorbidity with cannabis use disorders [38]. TRPV1 is a documented endocannabinoid receptor, a possible molecular correlate for these correlations. SLC5A7 is a neuronal choline transporter, and there is suggestive evidence from a meta-analysis of human genomics studies on BSDs that cholinergic transmission is potentially altered in patients with BSDs [3]. Protein levels of the human ortholog of CP are elevated in patients with schizophrenia, a related mental health disorder [39], and patients with Wilson’s Disease, a genetic disorder caused by dysfunction of an enzyme upstream of ceruloplasmin, have a higher incidence of BSDs than the general population [40]. Copy number variants in the human ortholog of the *Polr3c* gene have been reported in schizophrenic patients [41], and *Polr3c* was proposed as a schizophrenia candidate gene relative to a number of metabolites altered in plasma samples from schizophrenic patients. The POLR3C variant is at a conserved residue in the middle of an alpha helix, likely affecting the secondary structure of this protein. Altogether, we believe these variants imply fundamental systems-level construct validity for MSN as a mania model.

Though exome resequencing is useful to identify immediately compelling variants altering the functions of protein-coding genes, the technique has important limitations. Because it samples from the ~60 Mb of coding genome, a small subset of the ~3 Gb mouse genome, exome resequencing cannot identify alterations in gene regulatory regions that may occur far outside of the protein-coding region of the genome. Further, exome resequencing has limited power to identify copy-number variations, structural changes in genome structure that consist of insertions or deletions of large features. Though these caveats should be kept in mind, the structural genomic changes we have documented here represent initial behaviorally relevant genomic alterations in these mice that may have relevance to BSDs.

While structural genomic tools can find variants and generate hypotheses, validation is a more difficult, costly, and low throughput process. Functional characterization of these results is the next step in understanding the biological relevance of each variant. CRISPR allows the inexpensive generation of transgenic animal bearing these variants, making a knock-in of these MSN-unique variants to an unrelated strain like C57BL/6J animals a natural next step for functional assessment of each allele. This would allow for behavioral characterization of each variant in isolation from possible cis factors in unqueried parts of the genome. It would also provide a useful tool to measure the effects of these variants on protein function using assays specific to each gene of interest.

## Materials and methods

### Animals

Animal use was carried out in accordance with the recommendations in the Guide for the Care and Use of Laboratory Animals of the National Institutes of Health. The University of Wisconsin–Madison College of Letters and Sciences IACUC approved all animal protocols. All reasonable efforts were made to minimize animal suffering.

The animals in this study were randomly selected using a true random number generator (http://random.org) from breeding colonies of three mouse strains: the manic Madison (MSN) strain, the outbred Hsd:ICR (ICR) strain (Harlan Laboratories, Madison, WI, USA), and the Maternal Defense 2 (MaD2) strain. The MSN strain was derived from ICR animals over the course of approximately 15 years, going through 30 generations of selective breeding for high voluntary wheel running [42], then through 25 generations of selective breeding for maintenance of high maternal defense [43], and finally through a number of generations of random maintenance breeding before being characterized as manic [14]. MSN mice are available at the Mutant Mouse Regional Resource Centers as stock number 036809-MU. MaD2 is another ICR-derived strain with parallel selective breeding to MSN for high maternal defense, but that does not display a mania-like phenotype [44]. All animals from both the MSN and ICR strains were used in a previous behavioral study examining sexual dimorphism in the MSN phenotype [15], giving us a behavioral dataset of opportunity to use in this paper. The animals were group housed up until 48 hours prior to euthanization, when they were singly housed. All animals were euthanized by decapitation under deep anesthesia (inhaled 5% isoflurane) prior to tissue collection.

We exome resequenced a total of 3 MSN, 3 MaD2, and 5 ICR females and performed high resolution melt curve analysis of 32 MSN and 32 ICR samples from both males and females.

### Exome resequencing strategy

We utilized a total of 3 control strains. The outbred ICR strain, the strain from which the MSN progenitors were derived 15 years ago, is the behavioral reference against which we have compared the MSN animals in our previous work. As the source of the genetics leading to the MSN strain, ICR was the natural control strain for both finding new variants and for assessing the population allele structure leading to MSN. Additionally, the MaD2 mouse strain received parallel selective breeding for high maternal defense alongside the MSN progenitors, but does not show the same manic phenotype as MSN mice [14]. Finally, the C57BL/6J has been used as a control strain in studies of mania in inbred strains by other researchers [45]. As the strain sequenced for the reference genome we used, C57BL/6J was a tacit control.

The MSN breeding history and its phenotype relative to related and reference strains allows us to make a number of assumptions that reduce the amount of sequence necessary to find candidate variants: 1) MSN animals will be nearly fixed mismatching the C57BL/6J reference for explanatory variants; 2) ICR animals, which supplied important genetic variants, will show variability at these alleles, but ICR animals homozygous for variant alleles will be rare for variants important to the MSN phenotype; 3) MaD2 animals will show a similar allele pattern to ICR animals at important MSN alleles; and 4) important MSN alleles will change the biochemical function of the proteins their genes encode. From these assumptions, we resequenced the exomes of a sample of MSN animals and relevant control strains to find interesting protein-coding variants in the MSN genome. We followed up on the most interesting variants using a real-time PCR technology, high-resolution melt curve genotyping (HRM) [46], to measure differences in the population allele ratios of the most interesting variants between MSN and outbred ICR animals.

### DNA extraction, targeted exome capture, and deep sequencing

Since the genome is the same from all parts of the animal, we chose to extract liver DNA due to our ability to extract large quantities of clean genomic DNA from that tissue quickly and easily. After the animals were euthanized, we dissected and snap froze liver samples over dry ice, storing them at –80° C until DNA extraction. To extract DNA, liver slices were digested at 60° C overnight in a dry block heater in lysis buffer containing 50 mM Tris-HCl (pH 8.5), 5 mM EDTA, 10% SDS, 150 mM NaCl, and Proteinase K at 10 μg/mL (Thermo Fisher, Waltham, MA, USA). RNAse A/T1 mixure (Thermo Fisher, Waltham, MA, USA) was added at 2 μg/mL concentration an hour before organic extraction. DNA was extracted in 3 changes of phenol-chloroform-isoamyl alcohol mixture saturated in pH 8.0 Tris buffer, then cleared in 2 changes of chloroform before being precipitated in 2.5 volumes of ice-cold absolute ethanol. DNA pellets were desalted in two changes of ice-cold 70% ethanol, air dried, and dissolved in nuclease-free water.

After checking extracted DNA using a NanoDrop spectrophotometer (Thermo Fisher, Waltham, MA, USA) and gel electrophoresis for purity and quality, barcoded sequencing libraries was constructed using an Agilent SureSelect XT Whole Exome kit (Agilent, Austin, TX, USA) for mouse according to the manufacturer’s specifications. Briefly, gDNA was sheared to ~300 bp and ligated to adapters before hybridization to biotinylated ribobaits. Hybridized samples were captured and PCR-amplified with adapters containing sample-specific barcodes. The libraries were purified, quality checked using real-time PCR, then sequenced on a total of four HiSeq 2000 lanes across two runs. All sequencing procedures were performed by the University of Wisconsin-Madison Biotechnology Center’s Next Generation Sequencing Facility.

Bases were called, reads were demultiplexed, and FASTQ files were generated using CASAVA v. 1.8.2.

### Alignment, variant calling, and functional annotation

All analysis was done in OS X v. 10.9. Raw paired-end reads in FASTQ format were aligned to the GRCm38 genome used by the Sanger Mouse Genome Project (MGP) [47] with Bowtie 2 v. 2.1.0 [48]. Alignments were converted to BAM format, then pre-processed to update read groups, fix mate pair information, and mark PCR duplicates using Picard v1.101 (http://picard.sourceforge.net) running on Java v. 1.7.0_25. Reads were locally realigned around INDELs using the Genome Analysis Tool Kit (GATK) v. 2.7–4 [49] using the MGP INDELs VCF file aligned to GRCm38, then quality scores were recalibrated in GATK using the MGP SNPs VCF file aligned to GRCm38. Picard was used to fix mate pair information and merge BAM files from the same biological samples across lanes and then GATK was used to call SNPs and small INDELs as a VCF file. This VCF file was functionally annotated using SnpEff v. 3.3 [50] with the GRCm38.70 annotation.

The variants with the highest likelihood of uniqueness and relevance to the MSN phenotype are 1) mismatches to the C57BL/6J reference genome, 2) homozygous variant in all MSN animals, and 3) not homozygous variant in any ICR or MaD2 animals. We created a custom query in VarSifter v. 1.6, for only variants fitting these criteria. We filtered the results for functional changes to coding sequences, defined as: missense mutations (nsSNPs); insertions or deletions of codons in coding sequences; splice site variants; nonsense-mediated decay (NMD) variants; stop gain or loss variants; and frameshift variants. Variants meeting these criteria were queried with Ensembl’s VEP tool to predict likelihood of functional effects using SIFT [51].

Raw reads in FASTQ format are deposited in the NCBI Sequence Read Archive (SRA, http://www.ncbi.nlm.nih.gov/Traces/sra) under the SRA project accession number SRP040655.

### Functional enrichment

Functional enrichment utilized DAVID v6.8 [52]. Briefly, gene lists were converted into Ensembl Gene IDs using Ensembl’s Biomart tool for either the 87 genes in the long list of all variants with SIFT < 0.25 and the 24 genes in the short list (gene sets used for enrichment listed in S2 Table). Missing annotations were inserted after querying Ensembl. These genes were queried in the “Functional Clustering” tool, which identifies clusters of related functional annotations and rates each cluster using an enrichment score. Because the enrichment score is a -log_10_ transformation of a p-value, a score of 2.0 or greater is significant at the p < 0.01 level, which is the threshold we used for significance in this study. Functional annotation clustering utilized medium classification stringency, the default group of terms, and the default *Mus musculus* background.

### High resolution melt curve genotyping

To compare interesting alleles and their ratios in populations, we utilized a real-time PCR derived technique called high-resolution melt curve genotyping (HRM) [46]. This technique uses oligonucleotide primers flanking each side of a variant. After PCR amplification, the dissociation temperature of an amplicon is measured using a fluorescent dsDNA binding dye like EvaGreen while increasing temperature at 0.2° C increments. Subtle variations in dissociation temperature are associated with each genotype; HRM is sensitive enough to detect even A/T and T/A transversions [46].

We used HRM on 32 MSN and 32 ICR samples, randomly selected and balanced between males and females. Each HRM assay was calibrated to the sequencing results, allowing us to find genotypes for unsequenced samples. Since 3 MaD2 animals were sequenced, we performed HRM on these samples to aid calibration. PCRs were performed using 10 μL reactions with 4 ng of template, 500 nM concentrations of each primer, and a 1X reaction mixture of SsoFast EvaGreen Supermix (Bio-Rad, Hercules, CA, USA). PCRs were performed using a CFX-96 Touch real-time thermal cycler with fluorescence collected using CFX Manager v. 2.1 software (Bio-Rad, Hercules, CA, USA). Initial amplifications utilized a two-step hot start protocol with an initial 98° C dissociation step for 30 seconds, then 40 cycles of a 2 second 98° C dissociation step followed by 15 seconds at an empirically-determined oligonucleotide-specific annealing temperature. Fluorescent signal was measured in real-time during PCR, and all PCRs had a Cq between 20 and 30 as recommended by the manufacturer for HRM. After a 1 minute 98° C step and a 1 minute 70° C step, 10 second dissociation (melt) steps occurred at 0.2°C increments between 70° C and 95° C. Fluorescent signal was collected at each dissociation step, and dissociation curves were normalized and analyzed by statistical clustering for genotype using Precision Melt Analysis v. 1.2 (Bio-Rad, Hercules, CA, USA).

Though HRM genotyping does not have a published standard like the MIQE [53], we used the MIQE as a guide for reproducibility. S4 Table contains much of the information required by the MIQE standards including information about oligonucleotide primer sets and the annealing temperatures we used.

### Statistical analysis

All statistical analyses were performed in R version 3.0.2 for OS X x86-64. Deviations from Hardy-Weinberg equilibrium were tested using a chi-squared test with the pchisq function. Fisher’s Exact tests for allele ratios between strains were performed using the fisher.test function. False discovery rate correction utilized the p.adjust function with the Benjamini-Hochberg method [54]. ANOVAs were performed with genotypes on previously collected behavioral data from our previous work [15] that were transformed using the inverse of the square root as described previously [15]. Analysis of behavioral data utilized the anova function on linear models with inverse-square-root of the behavior for the 32 ICR animals in the HRM experiment as the dependent variable. Genotype at various loci as assessed by HRM, sex, and the interactions between sex and genotype were the independent variables for these ANOVAs.

## Supporting information

S1 Table Table of 119 variants meeting the criteria of being mostly fixed in MSN, variable in ICR and MaD2, and likely to change protein coding or splice variation. Table includes information about prior differential expression in our previous microarray work [16] as well as manual annotations about hypothesized variant consequences.
(XLSX)

S2 Table Enrichment results from DAVID. A) Functional enrichment clusters for the 87 genes with SIFT scores < 0.25. B) Functional enrichment clusters for the 24 genes on the short list. C) Gene IDs used as input for DAVID enrichment.
(XLSX)

S3 Table HRM genotyping results including genotypes and allele counts for each mouse strain of interest and relevant statistical tests. A) Explanation of genotype and allele types used in table. B) Raw HRM results with behavioral data included. C) Allele ratios and Fisher’s Exact tests. D) Genotype ratios and and Hardy-Weinberg equilibrium calculations.
(XLSX)

S4 Table MIQE-compliant HRM genotyping assay table including primers, primer annealing temperatures, and reaction conditions necessary to reproduce HRM analysis. Table includes: A) Experimental design, B) Tissue sample information, C) Nucleic acid extraction information, D) PCR target information, E) PCR oligonucleotide information, F) PCR protocol information, G) PCR validation information, H) Data analysis information, and I) Primer sequences.
(PDF)
