# Supplementary material for: Genomic variants in an inbred mouse model predict mania-like behaviors
Source: PLoS One. 2018 May 16;13(5):e0197624. doi: 10.1371/journal.pone.0197624 (PMC5955540; doi:10.1371/journal.pone.0197624)
Supplement: S4 Table — Table includes: A) Experimental design, B) Tissue sample information, C) Nucleic acid extraction information, D) PCR target information, E) PCR oligonucleotide information, F) PCR protocol information, G) PCR validation information, H) Data analysis information, and I) Primer sequences. (PDF) [file pone.0197624.s004.pdf]

S4 TABLE: HRM METHODS

S4A – Experimental design

Definition of experimental and control groups	Experimental: Madison (MSN) mouse strain. Control: outbred hsd:ICR (ICR) and Maternal Defense 2 (MaD2) mouse strains.
Number in each group	32 MSN, 32 ICR, and 3 MaD2. MSN and ICR mice were split evenly between male and female. All MaD2 mice were female.
Assay carried out by core or investigator's laboratory	Carried out in investigator's laboratory.
Authors' contributions to qPCR section	Michael C. Saul: all molecular work, half of dissection work, writing.<br>Sharon A. Stevenson: half of dissection work.<br>Stephen C. Gammie: bred mice, provided funding and lab space, writing.

Table S4B – Tissue Samples

Description	Fresh frozen liver.
Volume or Mass of sample	See table S4.10.
Dissection Type	Gross dissection of liver slices.
Processing Procedure	Animals were euthanized by decapitation under isoflurane anesthetic. Their brains and pieces of their liver were immediately dissected.
If frozen, how quickly?	Samples were flash frozen over dry ice upon dissection.
Sample storage conditions	Samples were stored at -80° C prior to DNA extraction.

Table S4C – Nucleic Acid Extraction

Procedure and/or instrumentation	Liver tissue was disrupted by enzymatic and chemical digestion overnight in a dry block heater. DNA was isolated in phenol-chloroform-isoamyl alcohol, cleared with chloroform, precipitated in ethanol, desalted, and dissolved in nuclease-free water.
Name of kit and details of any modifications	No kit was used.
Sources of additional reagents used	Proteinase K (Thermo-Fisher, catalog number EO0491)<br>Phenol-Chloroform-Isoamyl Alcohol in Tris buffer, pH 8.0 (Sigma-Aldrich, catalog number P2069)<br>Chloroform (Acros Organics, catalog number AC42355-0250)<br>Ethanol (Fisher Scientific, catalog number BP2818-500)
Details of RNase treatment	RNase treatment for 60 minutes during tissue digest with mixed RNase A/T1 (Thermo-Fisher, catalog number EN0551)
Contaminaion assessment of input DNA	NanoDrop curves used to assess presence of presence of protein, salt, and organic contaminants. All curves indicated clean samples.
Nucleic acid quantification	Nucleic acids quantified by A_{260} from the NanoDrop spectrophotometer.
Instrument and method of nucleic acid quantification	NanoDrop spectrophotometer, absorbance at 260nm.
Purity (A_{260}:A_{280})	All samples had A_{260}:A_{280} ratios between 1.8 and 2.2.

Table S4D – PCR Target Information

Gene symbol	See table S4.9.
Consequence	See table S4.9.
Location of amplicon	See table S4.9.
Amplicon length	See table S4.9.
In silico specificity	All primers screened for specificity using NCBI Primer-BLAST.
Sequence alignment	Aligned in NCBI Primer-BLAST.
Location of each primer in genome	See table S4.9.

Table S4E – PCR oligonucleotides

Primer sequences	See table S4.9.
Location and identity of any modifications	No modifications.
Manufacturer of oligonucleotides	UW-Madison Biotechnology Center DNA Synthesis Facility.
Purification method	Standard desalting and lyophilization.

Table S4F – PCR protocol

Complete reaction conditions	Bio-Rad SsoFast EvaGreen Super Mix (catalog number 172-5204) used according to manufacturer specifications without modification.
Reaction volume and amount of cDNA/DNA	10 μL reactions: 1 μL DNA at 4 ng/μL concentration used per reaction.
Primer Concentration	500 nM forward and 500 nM reverse primer for all primer sets.
Mg^{2+}concentration	3.0 mM $MgCl_2$.
dNTP concentration	200 μM each of dATP, dTTP, dCTP, and dGTP. 800 μM dNTP total.
Polymerase identity	Bio-Rad SsoFast Taq Fusion Polymerase.
Polymerase concentration	Proprietary concentration.
Buffer identity and manufacturer	Bio-Rad qPCR buffer provided with SsoFast EvaGreen Supermix.
Exact buffer chemistry	Proprietary composition.
PCR additives used	No additives used.
Manufacturer of plates and catalog number	Bio-Rad Hard Shell® 96-Well Skirted PCR Plates, Low-Profile (catalog number HSP-9601).

Complete thermal cycling parameters	Incubation stage: 30 s at 98° C. Cycling stage: 40 cycles, 2 steps: 2 s at 98° C, 15 s at annealing temperature (see table S4.10 for the specific annealing temperature used with each primer set).
Reaction setup	Manual using Eppendorf adjustable volume pipettes.
qPCR instrument	Bio-Rad CFX-96 Touch

Table S4G – PCR validation

Evidence of optimization	Prior to HRM analysis, we tested each primer set at an estimated annealing temperature based upon Primer-BLAST's implementation of the SantaLucia 1998 thermodynamic parameters. These temperatures provided adequate amplification for HRM genotyping in every case.
Specificity	Stringent *in silico* testing of primers prior to qPCR using Primer-BLAST, dissociation curve test of specificity *in vitro*.
C_q of NTC	C_q > 40 for all NTCs for all genes.

Table S4H – Data analysis

HRM analysis program	Bio-Rad Precision Melt Analysis.
Method of C_q determination	Used Bio-Rad's CFX Manager software.
Outlier identification and disposition	Our experiments contain no outliers, kinetic or otherwise.
Results from NTC	All NTCs showed no amplification.
Description of normalization	Data normalized for baseline fluorescence.
# of technical replicates	2 PCR technical replicates.
Statistical methods for results significance	Fisher's exact test with FDR correction.
Software (source, version) of stats	Bio-Rad CFX Manager v. 2.1; Precision Melt Analysis v. 1.2; R v. 3.0.2.

Table S4I – Primers

Gene + Consequence	*Primer Sequence (5'-3')*	*Product Length*	*Variant Location + Identity (mm10)*	*T_m*
Npas2 L481P	F: GCGACCTCACAAAGCAACT<br>R: CTGGAGCAGGTGGACTCTG	69 bp	chr1:39336044 T/C	60° C
Cp C712G	F: CAACTAATGCTCGCTTTGTCC<br>R: TTCCCTACTCACCTTGCTGT	190 bp	chr3:19980665 T/G	59° C
Hltf H22 NMD	F: TTCGCAGTCTGTCCAGTATG<br>R: GGAATTCAAAATGTGGCAAGAAAG	80 bp	chr3:20058944 C/T	58° C

Polr3c T268M	F: GCTAGAGGGAGTGGTGATCT R: TTTTCAGACAAGCAGCGAGA	67 bp	chr3:96719304 G/A	59° C
Cad R1608H	F: ATTTTGAAACCTGGCCTGC R: CTTCCGAGCCACATGACATAT	89 bp	chr5:31073236 G/A	58° C
Lpcat2b R112H	F: GGAGGAAACATCTGATAAAGTCG R: TAACCAGGAACCCAGCAAAA	95 bp	chr5:107433141 G/A	58° C
Ces1b Y118H	F: TTTTGTCAAATCGGCAGGGC R: GCATACCTCCCCTGTTTTCC	68 bp	chr8:93076109 A/G	59° C
Smarca4 R351Q	F: TGCCACCACAAACACAGTC R: TCTCCACAGGGTCAAGGC	120 bp	chr9:21637471 G/A	60° C
Trpv1 P14A	F: TTCAGTGAATATAAGGGCAGTGT R: CTGGTATCGAATCCAGGGAGG	69 bp	chr11:73240601 C/G	61° C
Nrac R28W	F: TGCCATCCAACCAGAGAGAA R: GTACCCTCTCAGTCCTGGAA	69 bp	chr12:112495085 C/T	59° C
Slc5a7 R38H	F: AGGACAAAGGCAATGTGTGAT R: TTCTGGTTGGAATATGGGCTG	195 bp	chr17:54297024 C/T	58° C
